# Optical properties and pulse shape discrimination in siloxane-based scintillation detectors

**DOI:** 10.1038/s41598-019-45307-8

**Published:** 2019-06-24

**Authors:** T. Marchi, F. Pino, C. L. Fontana, A. Quaranta, E. Zanazzi, M. Vesco, M. Cinausero, N. Daldosso, V. Paterlini, F. Gramegna, S. Moretto, G. Collazuol, M. Degerlier, D. Fabris, S. M. Carturan

**Affiliations:** 1 INFN, Laboratori Nazionali di Legnaro, Viale dell’Università 2, 35020 Legnaro, Padova Italy; 2 Department of Physics and Astronomy “Galilei”, University of Padova, Via Marzolo 8, 35100 Padova, Italy; 3 INFN – Sezione di Padova, Via Marzolo 8, 35100 Padova, Italy; 4 INFN - Trento Institute of Fundamental Physics and Applications (TIFPA), Via Sommarive 14, I-38123 Povo, Trento Italy; 5 Department of Industrial Engineering, University of Trento, Via Sommarive 9, Trento. Povo, 38123 Povo, Trento Italy; 6 Department of Computer Science, University of Verona, 37134 Verona, Italy; 7 Science and Art Faculty, Department of Physics, Nevsehir Haci Bektas Veli University, Nevsehir, Turkey; 8 KU Leuven, Instituut voor Kern- en Stralingsfysica, 3001 Leuven, Belgium

**Keywords:** Fluorescence spectroscopy, Characterization and analytical techniques, Experimental nuclear physics, Design, synthesis and processing

## Abstract

The possibility to detect fast neutrons as a distinct signal from that one of γ-rays background is surely of great importance for several topics, spanning from homeland security to radiation monitoring in nuclear physics research plants. Nowadays, Helium-3 based detectors are extremely expensive, while the use of large volume liquid scintillators presents serious concerns related to spillage risks and waste disposal. A very attractive alternative is the use of commercially available solid scintillators, which exploits an aromatic polymer matrix entrapping very high loadings of primary dye, thereby enabling the use of pulse shape analysis (PSA) to discriminate between fast neutrons and γ-rays. In this work, we analyse in detail the optical features of a solid scintillator composed by polymethylphenylsiloxane (PMPS) as base polymer loaded with moderate amounts of 2,5-diphenyloxazole (PPO). Furthermore, fluorescence decay kinetics have been correlated to the observed pulse shape discrimination capabilities of this radiation and thermally resistant scintillator, whose performances have been discussed in terms of conformational features and excimers formation revealed by the optical analyses.

## Introduction

Neutron detection through a simple and reliable methodology is highly desirable both in basic nuclear and particle physics experiments as well as in several applications. To cite some examples, neutron detector arrays can be used as multiplicity filters^1,2^, or as spectrometers^3,4^, in nuclear physics experiments. In particle physics, neutrino and anti neutrino detection is pursued by using very large volumes of scintillating materials, as in the case of SNO+ ^5^ and BOREXINO^6^, very recently plastic scintillating tiles with embedded waveshifting fibers were also used to fabricate Shashlik calorimeters to detect muons and hadron showers^7^. Among the applications it is worth mentioning the prevention of terrorism by monitoring neutrons emission from traveling goods^8^, avoiding spillage of contaminated liquids by detecting fission signatures from fissile materials in shielded containers^9^, neutrons dosimetry in potentially hazardous environments such as nuclear plants^10^, whereas metal recycling industry strongly needs monitoring of radioactive materials in scrap feeds^11^.

One of the strictest requirements when dealing with neutron detectors is their capability to discriminate fast neutrons from γ-ray background. Other ideal features for a fast neutron detector are high detection efficiency, long lasting performances, ease of processing and manipulation and low costs of production. For solid materials, the possibility of production in different size and shapes is commonly appreciated. Nowadays, some previously neglected issues should be addressed, such as the low toxicity levels of the chemical components and their environmental-friendly plus economically-acceptable disposal.

The most frequently used scintillators with fast neutron-γ discrimination capability are based on liquid systems like EJ-301^12^ and BC-501A^13^, where the solvent is a benzene parent compound, whose toxicity levels are among the highest ones in the class of chemicals.

Recently, less hazardous solvents with reduced vapour pressure at room temperature, higher flash-point and, in turn, minor health risk, have been proposed as dissolving media for scintillation cocktail, as in the case of EJ-309^12^. Among them, linear dialkylbenzene (LAB)^12–15^, and diisopropylnaphthalene (DIPN)^16^ are the best known.

Undoubtedly, significant improvement has been achieved as related to safety concerns by substituting pseudocumene with LAB or DIPN; nevertheless, the problem of waste disposal or liquid spillage is still a major critical point, owing to the large volumes needed to perform efficient fast neutron/γ-rays detection.

In this paper we focus on the design, production and characterization of a solid, siloxane-based scintillator to be applied for fast neutrons and γ-rays detection. To the best of our knowledge, the capability of a siloxane-based scintillator to discriminate particles on the basis of the different scintillation light pulse shape is demonstrated herein for the first time. Moreover, a correlation study between the optical features of the hybrid polysiloxane focusing on excimers concentration and enhanced scintillation pulse shape changes for different impinging particles is reported.

## Background Concepts

In organic systems, the scintillation process is based on the energy transfer between aromatic groups of the either solid or liquid matrix and the dispersed dye molecules characterized by high quantum efficiency. In fast neutrons and γ-rays detection, the particles actually measured by the scintillators are recoil protons from fast neutrons and recoil Compton electrons from γ-rays. Protons have a linear energy transfer (LET) much higher than electrons, producing a high density of ionization events along their path. The higher the ionization density, the higher the rate of formation of long-living triplet states (T_1_), as a result of recombination of ionized dye molecules with electrons^17^.

Two nearby T_1_ molecules can undergo triplet-triplet annihilation (TTA) to give a ground S_0_ and an excited S_1_ singlet state that in turn decays with the emission of a delayed photon^17–19^.

This *delayed fluorescence* has characteristic times of the order of tens of ns, with respect to the prompt emission, whose time scale is 1–2 ns, maintaining the same spectral response. The intensity of the component with longer lifetime increases with the concentration of triplet states, therefore recoil protons give rise to scintillation pulses with a more intense long-living component with respect to Compton electrons, thus allowing their discrimination through pulse shape discrimination (PSD) techniques.

The best algorithm to achieve the maximum discrimination is still a lively topic of research, especially using the new digital acquisition systems^20,21^, where the off-line signal processing can even make use of complex mathematical expressions.

Since TTA is a bimolecular short range process (less than 1 nm), in liquid scintillators this interaction can be easily accomplished as a *diffusion controlled* mechanism, as observed by many authors^17,18,22^. On the other hand, in plastic polymers such as polyvinyltoluene or polystyrene, TTA mechanism is hindered due to the stillness of the molecules in the rigid medium.

Recently, a plastic scintillator based on a polyvinyltoluene (PVT) matrix with high amounts of 2,5 diphenyloxazole (PPO), up to 30 wt%, as primary solute and traces of 9,10 –diphenylanthracene (DPA) as a waveshifter has been prepared by Zaitseva and co-workers^20^. These concentrations guarantee the proximity of fluorescent molecules, such to promote overlapping of their orbitals, while the impinging radiation leads to the TTA mechanism by directly ionizing the primary dye. Then, the final singlet excited states, which could give rise to delayed fluorescence, transfer their energy to the secondary dye.

It has been demonstrated that in this commercially available scintillator (EJ-299-33A, Eljen Technology) optimal PSD capability can be obtained, reaching remarkably good performances as compared to the traditional liquid systems^23–25^.

Nonetheless, the long-term stability of the final highly PPO loaded scintillator is still under investigation^19^. In fact, the massive presence inside the polymer network of the extraneous compound can be detrimental to the overall mechanical and thermal resistance, leading to deformation and deterioration with time of the detector. Very recently, Zaitseva and co-workers published new data related to an upgraded version of EJ-299-33A, where light transmission and mechanical stability in time have been improved^26^.

Another possibility to enable PSD in a solid scintillator can be explored by favouring the formation of primary dye clusters, with specific conformation to harvest energy from the matrix, through Förster-type mechanism, and allow the access to T_1_ states in close proximity to promote TTA. In this framework, the particular structure of polysiloxanes can be exploited in order to enable PSD without forcing the load of primary dye beyond the limit where irreversible changes in the basic features of the polymer are achieved.

**Figure 1:**
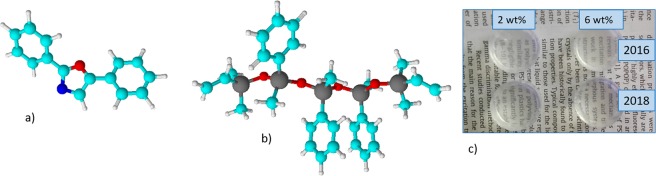
Structure of 2,5 diphenyloxazole (**a**) and of a fragment of PMPS (**b**) showing the spatial arrangement of phenyl rings as dyads; photos of the produced samples (1″ diameter, 1 cm thickness) in different years and different PPO wt% (**c**).

Polylsiloxane elastomers are known to be extremely versatile since the main backbone is constituted by the strong, flexible Si-O-Si bridging bond, whereas lateral substituents of the Si atom can be varied to properly functionalize the main chain, thus changing selectively some specific features, such as optical transparency, thermal resistance, refractive index and also gas permeability/selectivity. The PPO structure, shown in Fig. 1a, presents two lateral, non-polar phenyl groups bounded to a five-atoms ring comprising two heteroatoms, oxygen and nitrogen, which makes the central portion of the molecule quite polar and capable of hydrogen-bonding as acceptor. The solubility of PPO is negligible in polydimethylsiloxanes, but can be enhanced by using precursor siloxane resins with phenyl lateral substituents in the main structure. Moreover, the peculiar conformational structure of polymethylphenylsiloxane (PMPS) has been thoroughly studied^27,28^, and it was found that stable stacking of phenyl lateral rings can occur, where dyads and triads are optically active as excimers (Fig. 1b). Hence, those specific excimers forming sites in PMPS can be exploited to collect PPO molecules in such a quantity to form a sandwich-like microstructures, so that, in case of high amounts of T_1_ excited states, the rate of TTA is enhanced due to close proximity. Meanwhile, the amount of PPO needed to enable this phenomenon could be limited and not detrimental to the final properties of the polymer body, such as thermal resistance and optical transparency (Figs 1c and S1).

In this work we obtained a solid scintillator based on PMPS to combine the demonstrated optimal radiation resistance of the polysiloxane-based scintillators as compared to polystyrene and polyvinyltoluene analogues^29^ and the good light output under γ-rays and α particles as compared to traditional plastics^30^ with the intrinsic excimer forming sites presence of the PMPS itself, where the concentration controlled TTA mechanism can be promoted. Using this approach, the capability of the scintillator to discriminate between different particles can be enabled with minimal addition of primary dye, thus preserving the intrinsic and beneficial features of the base polymer, such as flexibility and optical transparency.

## Results

### Optical emission analyses and fluorescence kinetics profiles

Samples containing only PPO with concentrations up to the maximum value of 8 wt% were produced, since at higher concentration aggregates visible to the naked eye were observed, indicating a quite low solubility of the dye in PMPS. Nevertheless, up to 8 wt% of loading the samples display negligible effects on the optical transparency, as proved by the absorbance spectra reported in Fig. S1. Moreover, the poor solubility that induced us to add a low amount of PPO is not expected to be in contrast with the proximity of the luminescent molecules even at this low concentration level with respect to the one used in aromatic polymers like polyvinyltoluene. Hence, in order to analyze the distribution of PPO molecules into the PMPS matrix, we performed spectral and lifetime luminescence analyses on samples with different PPO concentrations, namely 1, 4, 6 and 8 wt%.

**Figure 2:**
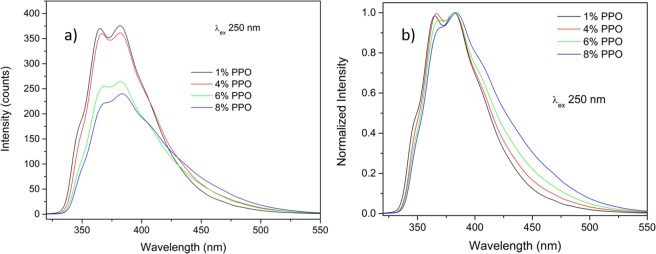
Fluorescence emission spectra (**a**) and normalized spectra (**b**) of PMPS loaded with different amounts of PPO.

Fluorescence emission spectra for samples with different PPO concentration are shown in Fig. 2a upon excitation at 250 nm. In Fig. 2b, the same spectra are reported by normalizing the intensity to the maximum value. The appearance of a long wavelength component at 420 nm for high PPO concentrations can be clearly noticed and its presence was connected with the formation of excimers between an excited state and a ground state PPO monomer molecule^31^. In fact, the same effect has been observed also for PPO in toluene solutions^32,33^, where for high concentration enhanced excimer emission and drop of monomer emission can be detected showing spectral shapes similar to the PMPS sample loaded with 6 wt% of PPO.

The PPO excimer formation and its features in terms of emission peak position and lifetime have been thoroughly investigated by several authors^32,34,35^, in function of dye concentration, solution temperature and solvent viscosity. At high dye concentrations, the molecules interaction probability increases, hence the formation of excimers is promoted, as can be seen from the gradually more pronounced intensity of the long wavelength component. On the other hand, this causes enhanced self-absorption and, in turn, concentration quenching. The latter effect can be clearly noticed from the overall emission intensity drop at increasing PPO concentration.

**Figure 3:**
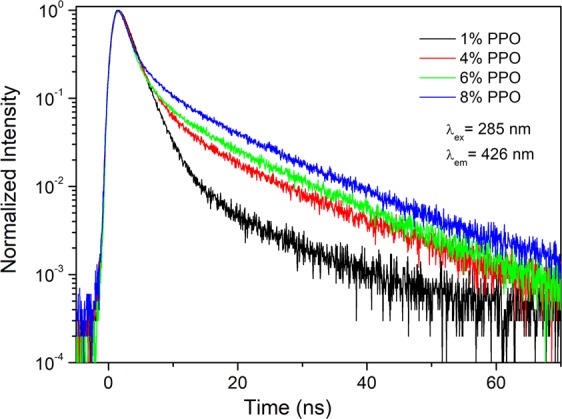
Fluorescence decay curves collected at 426 nm (λ_ex_ 285 nm) for samples with different amounts of PPO.

In Fig. 3 are shown the time-resolved intensity of the different samples collected at 426 nm, obtained with an excitation wavelength of 285 nm. As can be observed, the decaying part of the curves displays a double exponential shape, with a long lifetime component whose relative contribution increases with PPO concentration, in agreement with previously reported results on PPO dissolved in liquid siloxanes^36^.

The decaying part of the pulses was fitted using a double exponential decay function:

1 $$I(t)={A}_{f}{e}^{-t/{\tau }_{f}}+{A}_{s}{e}^{-t/{\tau }_{s}}$$

where τ_f_ and τ_s_ are the lifetimes of the fast and slow component, respectively, and *A*_*f*_ and *A*_*s*_ their amplitudes.

The fractional amplitude contribution of each component, either fast or slow, in the multi-exponential decay is calculated by the following relationships^37^.

2a $${f}_{fast}=\frac{{A}_{fast}{\tau }_{fast}}{{A}_{fast}{\tau }_{fast}+{A}_{slow}{\tau }_{slow}\,}$$

2b $${f}_{slow}=\frac{{A}_{slow}{\tau }_{slow}}{{A}_{fast}{\tau }_{fast}+{A}_{slow}{\tau }_{slow}\,}$$

In Table 1 the lifetimes with the error bars and the ratios between the fractional contributions of fast and slow components for the different concentrations are reported, whereas the fitting curves for each decay profile are reported in Fig. S2. It is worth to note that the higher error in the case of τ_s_ evaluation is due to the higher instrumental noise detected in the tail of the signal.

**Table 1:** Fitting parameters of the fluorescence decay profiles for each sample.

PPO	τ_f_ (ns)	τ_s_ (ns)	*f*_f_/*f*_s_
1 wt%	2.09 ± 0.01	14.9 ± 0.3	7.8 ± 0.7
4 wt%	2.20 ± 0.02	15.1 ± 0.9	2.5 ± 0.3
6 wt%	1.87 ± 0.02	13.1 ± 0.8	1.3 ± 0.1
8 wt%	1.72 ± 0.01	11.9 ± 0.6	0.60 ± 0.05

As can be observed, τ_f_ is around 2 ns, with the value decreasing with increasing PPO concentration. On the other hand, τ_s_ is in the range between 15 and 11 ns. By increasing the dye concentration, the ratio between the fractional contribution of fast and slow components decreases of one order of magnitude, thus pointing to an increasing contribution of the slower decay in the luminescence lifetime.

The fast and slow components are generally ascribed, respectively, to the regular singlet-singlet transition^32,38^, and to the emission of excimers formed by the coupling of very near PPO molecules^31,39^. The calculated values are in good agreement with other data previously reported for PPO liquid solutions^39,40^.

This comparison unambiguously demonstrates that in phenyl substituted polysiloxane the aggregation of monomers and formation of dyads of PPO is possible also at a relatively low concentration (<10 wt%). In fact, the added amount is not exceedingly high and does not imply the lack of intrinsic features of the polymer itself, which remains transparent, firm and though for several years, as can be seen in Fig. 1c.

Moreover, the presence of excimers clearly indicates that they are formed in the matrix even at 1 wt% of concentration. By evaluating the relative excimer amount through the *f*_*f*_*/f*_*s*_ ratio, where the fs factor is proportional to the concentration of excimer in the matrix, we can state that it increases of more than one order of magnitude from 1 to 8 wt% of concentration.

### Pulse shape discrimination

#### Energy calibration and scintillation light output evaluation

**Figure 4:**
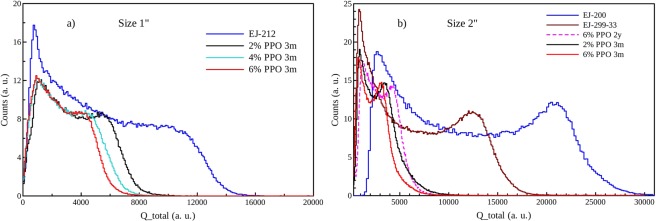
Pulse integral spectra of scintillators of different size a) 1″ and (**b**) 2″ upon exposure to ^137^Cs source. In (**a**) the response of samples produced 3 months before the analysis with increasing PPO wt% are shown.

Energy calibration in keV electron equivalent of the scintillators has been performed by exposing the samples to a ^137^Cs γ-ray source. The pulse integral (Q_total) spectra for the 1″ and 2″ samples with different concentrations of PPO are shown in Fig. 4. Due to the composition of the scintillators (low Z and low density) and to their dimensions, the main interaction occurring when γ-rays impinge on the detectors is Compton scattering, as can be observed from the spectra profile. The Compton edge (C.E.) can be used to convert the pulse integral from channels to energy (keVee), as described in refs^41,42^. Briefly, the pulse-height spectrum obtained by irradiation with ^137^Cs source is fitted by the Klein-Nishina distribution predicted on theoretical basis, modified by the experimental Gaussian energy broadening. Then, the obtained smearing factor is used to properly shift the obtained Compton maximum from the parabolic fit to the correct value of Compton edge.

The light output (L.O.) is proportional to the C.E. channel for each sample, and the relative scintillation yield was calculated as compared to commercial scintillators, namely EJ-212 or EJ-200, whose yield is known from manufacturer datasheets^12^. The obtained values and the characteristics of each scintillator in terms of composition, size and age of production are reported in Table 2.

**Table 2:** Dimensions and light output of the produced scintillators, estimated with respect to standard scintillators, using ^137^Cs γ-rays source. Indication of the sample age is given.

Sample	Size Ø [mm] × h [mm]	Volume [cm^3^]	% L.O.[*]
EJ-212	25.4 × 10	5.1	100
PMPS 2PPO (3 m)	25.4 × 10	5.1	56
PMPS 4PPO (3 m)	25.4 × 10	5.1	45
PMPS 6PPO (3 m)	25.4 × 10	5.1	42
PMPS 2PPO (2 y)	25.4 × 10	5.1	57
PMPS 4PPO (2 y)	25.4 × 10	5.1	51
PMPS 6PPO (2 y)	25.4 × 10	5.1	47
EJ-200	50 × 50	98	100
EJ-299-33A	50 × 50	98	62
PMPS 2PPO (3 m)	50 × 48	94	20
PMPS 6PPO (2 y)	50 × 44	86	22
PMPS 6PPO (3 m)	52 × 53	112	17

[*] Uncertainty on L.O. evaluation is around 10% as estimated by Stevanato *et al*.^42^.

It is worth to remember that EJ-200 and EJ-212 are formally the same type of plastic scintillator as far as the L.O. is concerned (in terms of % of anthracene it is 64% for EJ-200 and 65% for EJ-212, the only difference between the two formulations is the attenuation length, higher for EJ-200). In the case of small samples, the L.O. progressively decreases as PPO concentration increases. This is in agreement with previous observation of the emission intensity, reported in Fig. 2a, where reabsorption and concentration quenching phenomena induced by the high amount of dye lead to a remarkable emission drop.

For small samples (1″ diameter, thickness 10 mm), produced three months before the measurements (label 3 m in Table 2), the scintillator with 2 wt% PPO exhibits a L.O. of 56% respect to EJ-212. This value moderately decreases by increasing the PPO concentration, down to 45% for 4 wt% PPO and 42% for 6 wt% PPO. Samples produced two years before the measurements (label 2 y) with the same composition display comparable light output, thus demonstrating their high stability with time and weathering resistance. As for larger and thicker samples (2″), EJ-299-33A, the standard plastic scintillator for n-γ discrimination purposes, displays a L.O. of 62% respect to EJ-200 with the same size. As related to the scintillator with 6 wt% PPO and 2″ in diameter and thickness, aged 2 years, the L.O. is dramatically reduced to 22% of the standard EJ-200. Such a low value can be partially ascribed to the low attenuation length of polysiloxanes with respect to polystyrene and polyvinyltoluene based systems, as evidenced by transmission spectra reported in literature^43^. Interestingly, the same formulation but related to a sample aged only 3 months before the measure displays a slightly lower L.O. owing to the increased optical path of the object (53 mm versus 44 mm), thus proving that light losses due to reabsorption is a crucial factor. On the other hand, the lowering of the scintillation yield by increasing the sample size is a well-known phenomenon related to several parameters, namely self-absorption^44^, scattering loss at the surface^45,46^, proper wrapping^47^, coupling with the PMT^48^. Actually, the observed variation in light yield is quite high and some other phenomena could contribute in decreasing light collection at the PMT interface. In particular, the preparation of large size siloxane scintillators can be optimized as for flatness of the contact surface with PMT window, PPO dissolution level, which requires longer times than small samples, fine control of the Pt added catalyst, that should be as low as possible to minimize Pt nanoclusters formation with consequent yellowing. All those aspects are currently being considered in the production of new large size samples for a more extensive and systematic characterization.

#### Scintillation pulse analysis and charge integration method description

**Figure 5:**
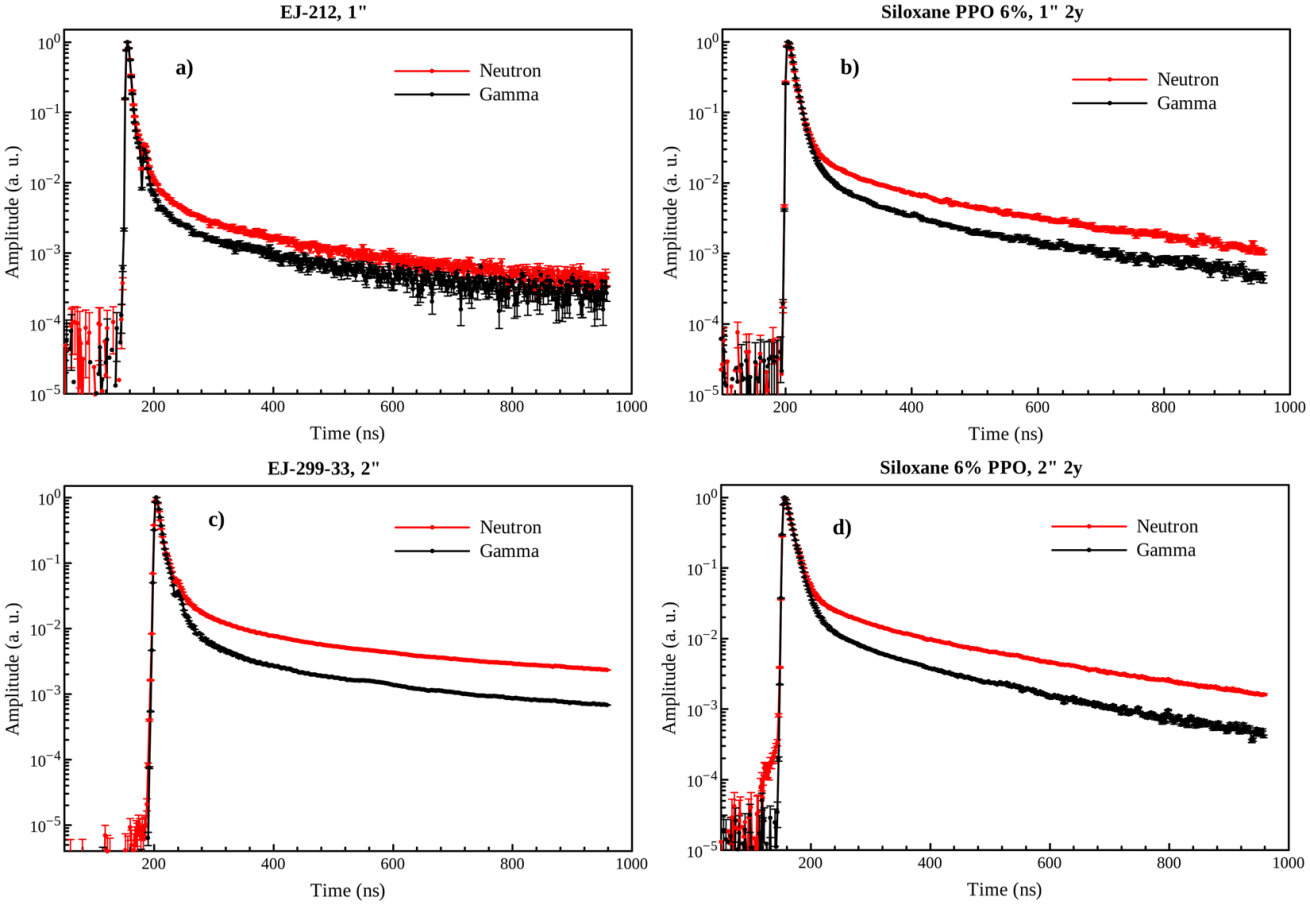
Scintillation pulses normalized on the pulse height obtained by averaging off-line waveforms for 1″ EJ-212 (**a**), 1″ PMPS 6 wt% PPO 2y (**b**), 2″ EJ-299-33 (**c**) and 2″ PMPS 6% PPO 2y (**d**).

The averaged signals of fast neutron and gamma events, for the synthesized 1″ and 2″ PMPS 6 wt% PPO based scintillators and for their corresponding standards of the same size, are shown in Fig. 5. The signals correspond to a deposited energy of 480 ± 10 keVee.

The waveforms displayed by the 6 wt% PPO loaded siloxane based scintillator either 1″ or 2″ in size clearly point to a feasible discrimination between fast neutrons and γ-rays, while the sample EJ-212 does not show, as expected, a remarkable divergence in the tail of the signal for different incident particles. Interestingly, the decay profiles in the case of EJ-299-33, the commercial standard for detection and discrimination of fast neutrons and γ-rays, are quite similar to those collected for the sample 6 wt% PPO, thereby indicating a promising behaviour as far as PSD capability is concerned.

In order to select the optimal parameters to be set in the signal processing to achieve the best particle discrimination, the most suitable time windows for integration of both the fast component and the slow one have been selected empirically, by optimizing the separation between γ-rays and fast neutrons signals. The signal integration method proposed by Cester and co-workers^24^ has been used in the following analysis, i.e. the pulse shape parameter (PSP) is estimated by comparing the slow part of the time decay profile (Q_tail) with the total integral of the signal (Q_total). Hence, the PSP is evaluated as follows

3 $$PSP=\frac{{Q}_{total}-{Q}_{fast}}{{Q}_{total}}=\frac{{Q}_{tail}}{{Q}_{total}}$$

Aiming at a more clear characterization of the n-γ discrimination capability of the tested scintillators and at a reliable comparison between different composition, size and age, we calculated the figure of merit (FoM) in the conventional form:

4 $$FoM=\frac{{\rm{\Delta }}}{{\delta }_{n}+{\delta }_{\gamma }}$$

where *Δ* is the separation between the PSP centroids of the neutron and γ distributions, and *δ*_*n*_ and 𝛿_γ_ are the full-width at half-maximum (FWHM) of the neutron and *γ* peaks, respectively.

**Figure 6:**
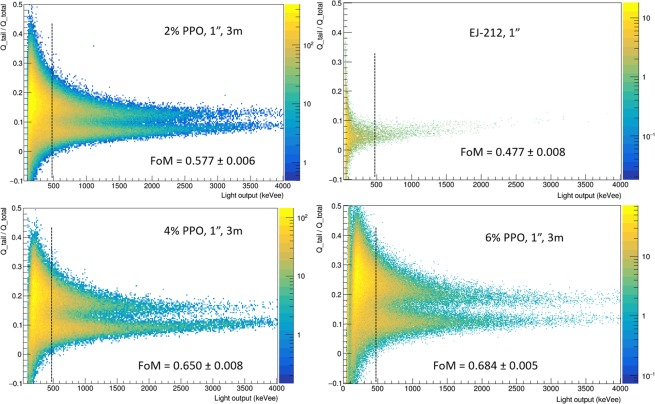
Two dimensional plots of PSP versus light output for the 1″ samples (aged 3 m) with different wt% PPO and of EJ-212 under irradiation with Pb shielded ^252^Cf source; the evaluated FoM at the threshold 480 ± 75 keVee, shown in each plot as a dashed line, is also reported for each scintillator and listed in Table 3.

In Fig. 6 we report the two dimensional spectra of PSP versus the light output in keVee for the commercial standard EJ-212 and for three 1″ size samples with increasing primary dye loading. The capability of the siloxane-based samples to discriminate different particles is clearly evident, also for low PPO concentrations, on the basis of the bare application of the described method. On the other hand, EJ-212 does not display a clear distinction between fast neutrons and photons, confirming the intrinsic inability of this plastic scintillator for particles discrimination.

**Figure 7:**
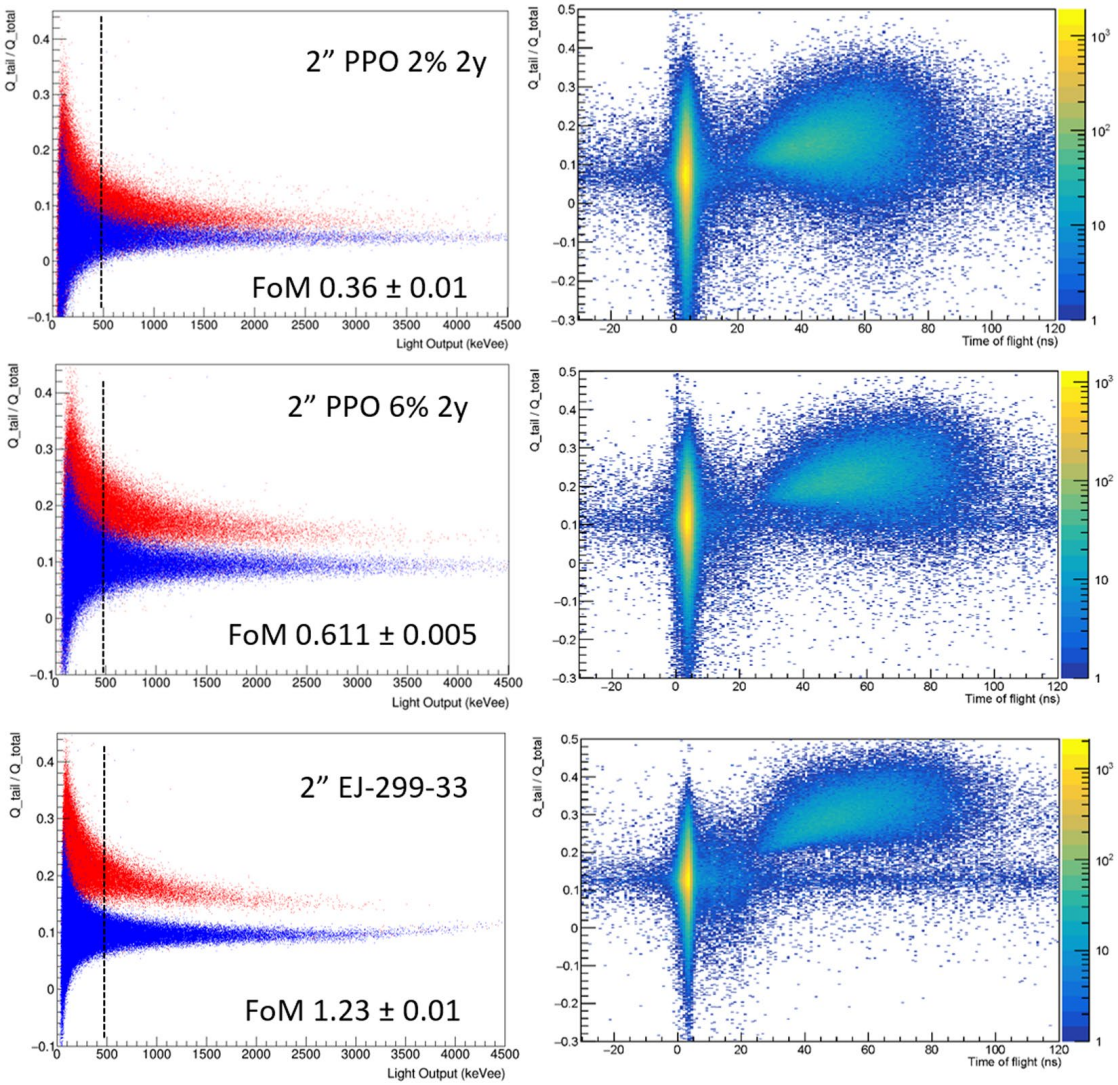
2D plots showing the PSP versus light output (left panel), with γ-rays and fast neutrons events labelled as blue and red dots, respectively. Contour plots of PSP versus TOF (right panel), showing the distribution of γ-rays, fast neutrons and uncorrelated background events. The FoM estimated for each sample at 480 ± 75 keVee is also reported.

The optimal performance of the synthesized detectors as for discrimination in mixed radiation fields can be even more appreciated with 2″ samples, whose PSP vs light output is reported in Fig. 7 (left panel). Using the TOF discrimination, γ-rays and fast neutrons are labelled as blue and red dots, respectively, in the PSP vs light output plots. In the same figure, the right side panel shows the scatterplot of PSP vs TOF, highlighting the correlation between the PSP and TOF parameters. In these figures, the two clearly visible loci are assigned to γ-rays, lower TOF values, and neutrons, higher TOF values. The flat horizontal distribution is originated from uncorrelated background events. It is worth to observe that for the standard plastic scintillator for n-γ discrimination, EJ-299-33, the PSP region with values higher than 0.15 can be assigned to neutrons and the events in this range which are indeed induced by γ-rays are limited to a small fraction. As for the 2″ PMPS scintillator with 2 wt% PPO, it does not show a clear distinction in PSP for different particles, pointing to almost no discrimination on the basis of the sole PSP. On the other hand, the sample loaded with 6 wt% PPO displays a good discrimination performance: the maximum of γ-rays induced events being centred at PSP 0.15 and the neutrons ones at PSP 0.22. Moreover, misclassification of the particles is limited as demonstrated by the TOF fingerprint of neutrons and γ-rays associated with PSP, where a clean separation is observed for this scintillator, though not as good as for EJ-299-33.

As for the FoM values reported in Table 3 for both 1″ (Fig. 6) and 2″ (Fig. 7) scintillators, small detectors display enhanced FoM for the same energy values than the larger ones, as already reported in literature^49,50^. Together with a lower yield, as previously stated, larger samples present a broader spread of the statistical distribution of detected scintillation photons as a function of time due to a wider distribution of paths and a higher number of reflections before reaching the photomultiplier tube. These effects lead to a broadening in the PSP distribution and, in turn, to lower values of FoM for a fixed threshold energy. As for the ageing study, samples with different ages display negligible variation in pulse shape discrimination performances, as can be seen in Fig. S3 and Table 3.

**Table 3:** Figure of Merit (FoM) values obtained using the parameters derived from PSP versus Light output plots at threshold 480 ± 75 keVee and applying Eq. 4.

Sample	Size Ø [mm] × h [mm]	Volume [cm^3^]	FoM
EJ-212	25.4 × 10	5.1	0.477 ± 0.008
PMPS 2PPO (3 m)	25.4 × 10	5.1	0.577 ± 0.006
PMPS 4PPO (3 m)	25.4 × 10	5.1	0.650 ± 0.008
PMPS 6PPO (3 m)	25.4 × 10	5.1	0.684 ± 0.005
EJ-299-33A	50 × 50	98	1.23 ± 0.01
PMPS 2PPO (2 y)	50 × 44	86	0.36 ± 0.01
PMPS 6PPO (3 m)	52 × 53	112	0.523 ± 0.005
PMPS 6PPO (2 y)	50 × 44	86	0.611 ± 0.005

## Discussion

As previously stated, the PSD in organic scintillators is related to the possibility of the doping dye molecules to be very close in order to promote the TTA phenomenon. This capability has been reached either in liquid scintillators, through the molecular diffusion, or in plastic systems with very high dye concentrations (up to 30 wt%)^20^.

In PMPS scintillators, studied in the present work, luminescence analyses demonstrated that PPO excited dimers molecules in very close proximity can be detected at concentrations as low as 1 wt% and that their amount increases with PPO concentration. This behaviour can be explained by taking into account the dissolution mechanism of PPO in the polysiloxane matrix.

Previous studies on PPO optical properties in different solvents^40^ evidenced that excimers are more readily formed in cyclohexane than in benzene solutions, owing to a lower affinity towards non aromatic media of the dye molecule. In the case of solid systems such as PMPS, the macromolecule presents both aromatic and non-aromatic side groups along in the chain. In particular, the conformation of siloxane chain with methyl and phenyl lateral groups has been thoroughly studied:^28^ the Si-O bond length and the wide angle of the Si-O-Si bridge that builds the polysiloxane backbone account for the high deformability of the chain itself and, in turn, for the formation of dyads and triads of facing phenyl pendant groups, thus complying with their mutual attractive interactions. This has been also observed by fluorescence spectroscopy on hybrids of polydimethyl-diphenylsiloxane and polyphenylmethylsiloxane, where the presence of dyads/triads as excimer forming sites was identified by their peculiar emission features^51^.

The presence of these phenyl stacking groups arranged in a sandwich-like conformation can effectively promote the localization of PPO molecules in preferential sites. In fact, PPO has a symmetric structure (see Fig. 1a), with the heterocycle in the middle portion and lateral phenyl groups and the molecule is almost planar with very small dihedral angles between the benzene rings as well as between benzene rings and oxazole ring^52^. In this scenario, specific sites where dyads and triads of phenyl pendant groups of PMPS are localized can easily attract PPO molecules, which gather in the sites promoting the formation of aggregates and, in turn, of excimers. Since negligible changes are seen in the optical transmittance of the samples through all the tested PPO concentrations (see Fig. S1), it can be deduced that in these sites the molecules do not form micro-precipitates, and in turn scattering centres, such to worsen the sample transparency.

The presence of excimers is clearly evidenced by the steady-state and time-resolved luminescence measurements reported above. In the monomer form, PPO gives rise to prompt fluorescence from singlet excited state with a fast decay of 2 ns and a spectrum peaked at 370 nm. When two PPO molecules are very close, the luminescence may occur from an excimer, which results from association of singlet excited and unexcited monomers, according to the following scheme^32^

$${{\rm{S}}}^{{\rm{1}}}+{{\rm{S}}}^{{\rm{0}}}\to {({\rm{SS}})}^{\ast }\to {{\rm{S}}}^{{\rm{0}}}+{{\rm{S}}}^{{\rm{0}}}$$

Excimers are characterized by a broad spectrum peaked at higher wavelengths with respect to the monomer and by a slower decay time. Both the features are observed to increase with the PPO concentration in the examined samples.

The formation of excimers is indeed related to the TTA mechanism under excitation with ionizing radiation (i.e. scintillation), since both processes occur with PPO molecules in close proximity.

As previously recalled, energy is transferred by the impinging radiation to the base material causing both ionization and excitation. The excitation energy is transferred by non-radiative, Förster type mechanisms to PPO^31,32,38^. Meanwhile, ionized PPO molecules recollect electrons forming triplet excited states leading to TTA and to delayed excited state transferring their energy to the secondary dye (LV in the present case). This dye, added in hundredths of PPO loading, plays the role to shift the final emission wavelength in the range of maximum responsivity of the photodetector and to increase the attenuation length^53^. Since triplet states are more populated in case of impinging particles with high LET and consequently high ionization density, TTA is favoured during the detection of protons or alpha particles with respect to gamma rays.

As a matter of fact, in liquid scintillating systems, where the molecular mobility is quite high, the TTA process can be effectively exploited to tag the presence of fast neutrons, owing to the appearance of the long-lived component of the light pulse. On the other hand, in plastic scintillators such as EJ-212 or EJ-200 the production of triplet states of primary dye molecules cannot be followed by annihilation, since those states are frozen inside the polyvinyltoluene matrix and their mobility is hindered.

In PMPS, the torsional freedom of the Si-O-Si bond leads to the formation of lateral phenyl groups stacks, with strong affinity towards the PPO molecule. Those sites become then active for the formation of excimers under excitation and, in turn, closely arranged triplet states. Therefore, PMPS is an optimal polymer matrix not only to dissolve the primary dye at reasonable level without compromising the transparency and the flexibility of the body, but also for enabling the TTA mechanisms to induce delayed fluorescence and unambiguously reveal the passage of fast neutrons over γ-rays background.

## Conclusions

We produced polymethylphenylsiloxane samples, added with low amounts of PPO as primary dye and a fixed amount of waveshifter, to obtain organic scintillators with the capability to discriminate between fast neutrons and γ-rays. This feature is related to the mechanism of triplet-triplet annihilation, which leads to delayed fluorescence and, in turn, to a longer decay time of the scintillation pulse in case of passage of particles with high ionization density, such as recoil protons from impinging neutrons. To enhance TTA, one should promote the diffusion and mobility or the close proximity of triplet states generated in primary dye molecules. A locally high concentration of triplet states of primary dye can be achieved in aromatic polymers, such as polystyrene or polyvinyltoluene, by adding a huge amount of PPO, though remaining below the solubility limit. On the other hand, the possibility to improve the local concentration of T^1^ excited PPO molecules, avoiding overloading the polymer itself, is very attractive, since it implies that the perturbation of the intrinsic properties of the matrix is minimized and the stability over time of mechanical and optical features is pursued. By exploiting the presence along the chain of PMPS of dyads of phenyl rings, it is possible to promote the aggregation of PPO molecules in those sites keeping the overall concentration quite low. The aggregation of PPO was evidenced by the formation of excimers, as demonstrated by fluorescence spectroscopy. The close proximity of PPO molecules makes possible the identification of fast neutrons over γ-rays, owing to the TTA mechanism giving rise to a variation in the shape of the scintillation light pulse with particles with different ionization density. Samples with PPO as low as 6 wt% display good n-γ discrimination performance, as compared with commercial plastic scintillator EJ-299-33, with preservation of their light output and discrimination capability over long periods of time. Optical transparency, flexibility and high light response are ascribed to the minimal perturbation induced by the dissolved additives, whose amount is low enough to preserve all the functional properties. This type of scintillator can be used with great advantages in environments where a remarkable mechanical stress occurs during the measurements and a soft and flexible material can offer outstanding benefits. Moreover, the well-known thermal resistance of polysiloxane can be exploited to produce scintillators able to withstand thermal gradients, hence further tests are ongoing to check the light output stability and discrimination capabilities over a wide range of temperatures. In summary, we presented for the first time a siloxane-based scintillator where the ability to discriminate fast neutrons and γ-rays has been demonstrated. Furthermore, the correlation between primary dye PPO excimers formation and the discrimination capability of the scintillator has been thoroughly investigated, on the basis of the chemical structure and conformation of the polysiloxane main chain.

## Materials and Methods

### Scintillators synthesis

Siloxane precursor resins containing phenyl groups along the main chain were used. In particular, polyphenylmethyl vinyl terminated siloxane (PMPS, Gelest PMV-9925) was used as base resin and mixed with the primary solute PPO in different weight ratios from 1 up to 8 wt%. Higher amounts of PPO in the resins were found to exceed the dye solubility limit, giving rise to precipitates. After complete dissolution of PPO, the waveshifting dye BASF Lumogen Violet (LV) was added (0.02 wt%). The mixture was left under stirring until a homogeneous and clear solution was achieved, then the Pt catalyst and the cross-linker resin were added and, after further 1 h of thorough mixing, the clear solution was outgassed in vacuum prior to casting in polyethylene cylindrical moulds. Further details on the synthesis of the siloxane scintillators can be found elsewhere^29,30^. The samples were extracted easily from the mould after 24 h of cross-linking reaction at 60 °C. One large size cylinder with diameter and thickness 50 mm (2 inches) was obtained with PPO 6 wt% and LV 0.02 wt% for comparison with EJ-299-33 and EJ-200 of the same size. Smaller samples (thickness 10 mm, diameter 1″) were also produced to evaluate the effect of increasing primary dye amount on pulse shape discrimination performances. The samples produced are highly transparent, firm and solid, though elastic. For comparison, a series of small size samples was prepared with PPO only, without LV addition, for the sake of clarity in fluorescence/excitation spectra. Excitation and fluorescence spectra of the polysiloxane based scintillators were recorded with a Jasco FP-6300 spectrofluorimeter, equipped with a 150 W Xe lamp. The bandwidth for both excitation and emission wavelengths was 5 nm.

### Emission spectra and time-resolved fluorescence analyses

Fluorescence lifetime measurements were performed with a Horiba Nanolog spectrofluorimeter, exciting the samples with 285 nm pulsed LED and recording the emission intensity in function of time at four different wavelengths: 361 nm, 385 nm, 426 nm and 500 nm. The LED pulse width is of 1.2 ns and the PMT rise time (R928P Hamamatsu) is 2.2 ns. The decay curves have been deconvolved taking into account of the instrumental response and the fittings were performed starting from the maximum of the pulse shape.

### Scintillation pulse acquisition, pulse shape parameter and time-of-flight analysis

**Figure 8:**
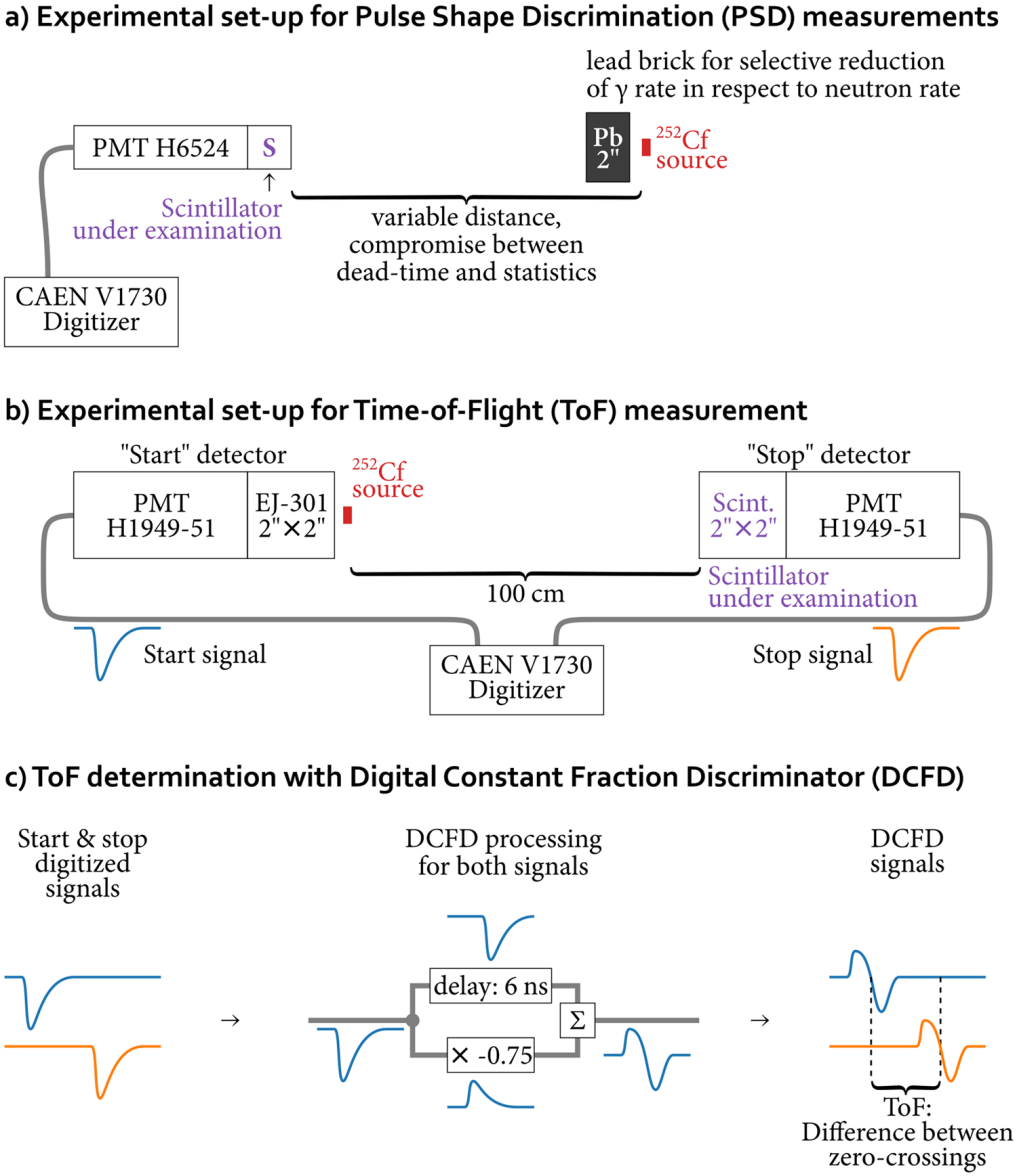
Schematic description of the adopted set-up for the evaluation of (**a**) discrimination capabilities on the basis of scintillation pulse shape and (**b**) Time-of-Flight response of the tested scintillators. The data acquisition system and processing for ToF measurements is reported in (**c**).

Scintillators were coupled to two different photomultipliers (PMTs) using a small amount of optical grease. Hamamatsu H6524 was used for the 1″ scintillators and Hamamatsu H1949-51 for the 2″ ones. The PMTs were operated at −1100 V and −1500 V respectively. Samples were exposed to a neutron/gamma field using a lead shielded ^252^Cf source (about 0.8 × 10^4^ neutrons/s) placed in front face of each sample, as shown in Fig. 8a. The distance between the source and the scintillator was varied for each sample, in order to get a suitable counting rate for a good statistic measurement in a reasonable period of time. The signals produced by the PMTs were fed into a VME CAEN V1730 14 bit 500MS/s digitizer^54^, allowing to record full waveforms of any single scintillation pulse. The pulse shape analysis has been carried out through the method proposed by Cester *et al*.^24^, thus defining two time intervals for integration of the scintillation pulse to derive the pulse shape parameter, as explained in more detail in the Results section.

On the other hand, with 2″ size scintillators, in order to unambiguously tag neutrons and γ-rays induced events on the scintillator, meanwhile estimating the efficacy of the adopted algorithm for PSD, the time-of-flight (TOF) technique was adopted. The TOF set-up consisted of a commercial EJ-301 liquid scintillator (2″ × 2″ cell) coupled to a PMT H1949-51 used as the “start” reference. The ^252^Cf source was placed just in front of the EJ-301 detector, while the triggering “stop” detector, i.e. the scintillator under study, was placed at a distance of 100 cm from the source, as schematized in Fig. 8b. Time resolution of a system of detectors similar to the one presented in this work was reported in ref. ^24^. and it was σ_τ_ ≈ 0.6 ns. The CAEN V1730 digitizer has a Digital Constant Fraction Discrimination (DCFD) embedded in the firmware which allows to perform very accurate on-line time measurements (see Fig. 8c for details). For fast pulses, it was found that the optimal values for the fraction and the delay of the DCFD were 75% and 6 ns.

The large difference in the velocity of photons and neutrons allows the classification of each particle on the basis of the difference in arrival time. The particle identification on the basis of TOF classifications can be exploited as a reference to evaluate the performance of the chosen PSD method, using a two-dimensional correlation plot between the measured pulse shape parameter, as described above, and the TOF.

## Supplementary information

supplementary information
