# Supplementary material for: Optical properties and pulse shape discrimination in siloxane-based scintillation detectors
Source: Sci Rep. 2019 Jun 24;9:9154. doi: 10.1038/s41598-019-45307-8 (PMC6591301; doi:10.1038/s41598-019-45307-8)
Supplement: Supplementary file 1 — supplementary information [file 41598_2019_45307_MOESM1_ESM.pdf]

Supporting Information

Optical properties and pulse shape discrimination in siloxane-based scintillation detectors

T. Marchi, F. Pino, C.L. Fontana, A. Quaranta, E. Zanazzi, M. Vesco, M. Cinausero, N. Daldosso, V. Paterlini, F. Gramegna, S. Moretto, G. Collazuol, M. Degerlier, D. Fabris, S.M. Carturan

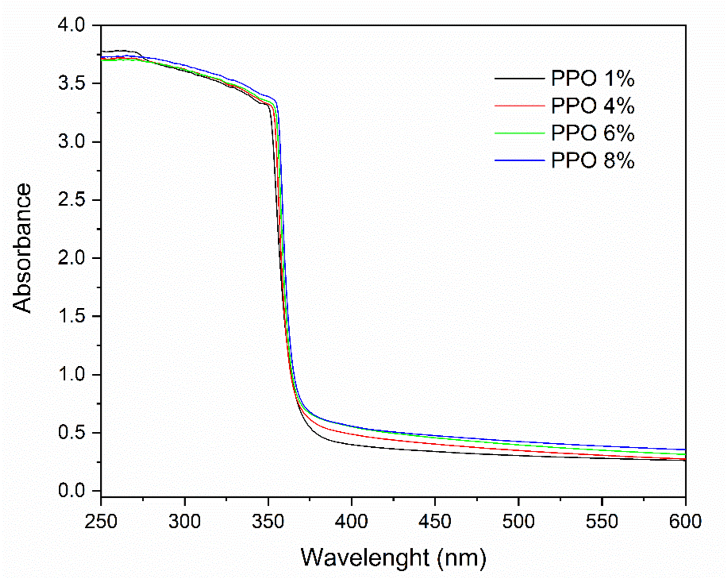

Figure S1. UV Vis absorption spectra collected for samples with increasing concentration of PPO and similar thickness, without Lumogen Violet. Photos of the same samples are reported in Figure 1c of the manuscript. The used UV-Vis apparatus is Jasco V-570.

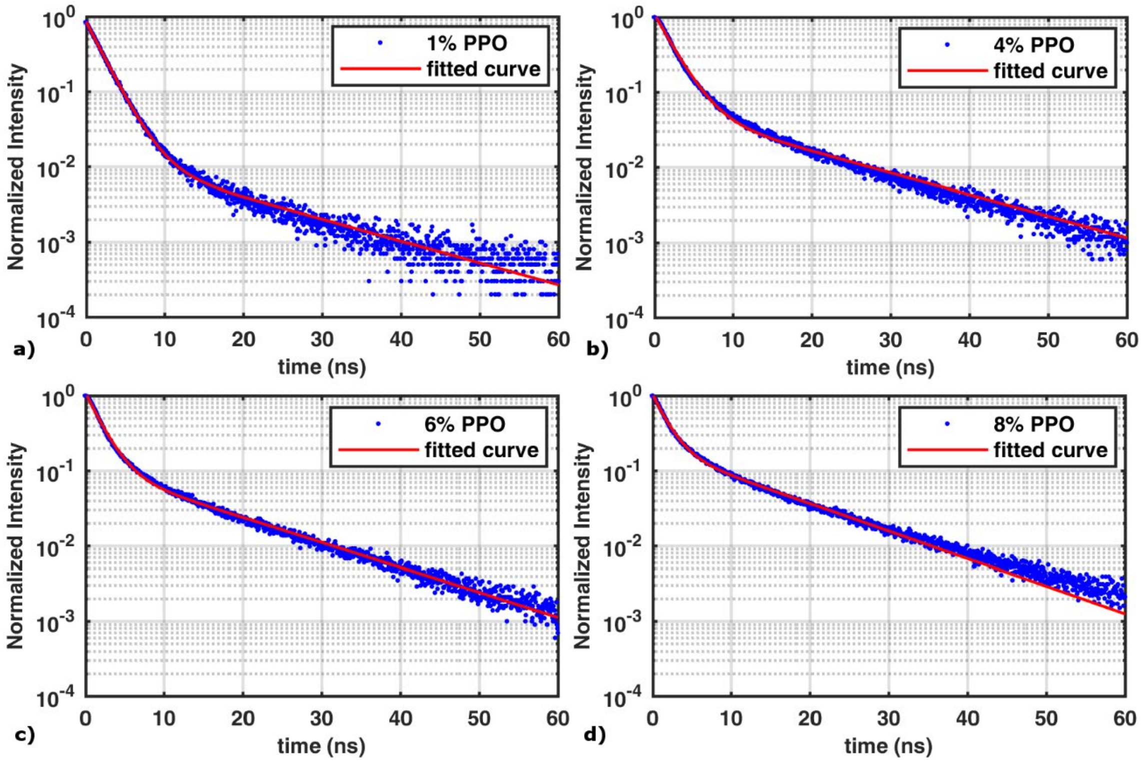

Figure S2. Fluorescence time decay profiles of samples with different PPO concentrations and relative bi-exponential fit (red curve). The emission signal is collected at 426 nm (λ_{ex} 285 nm).

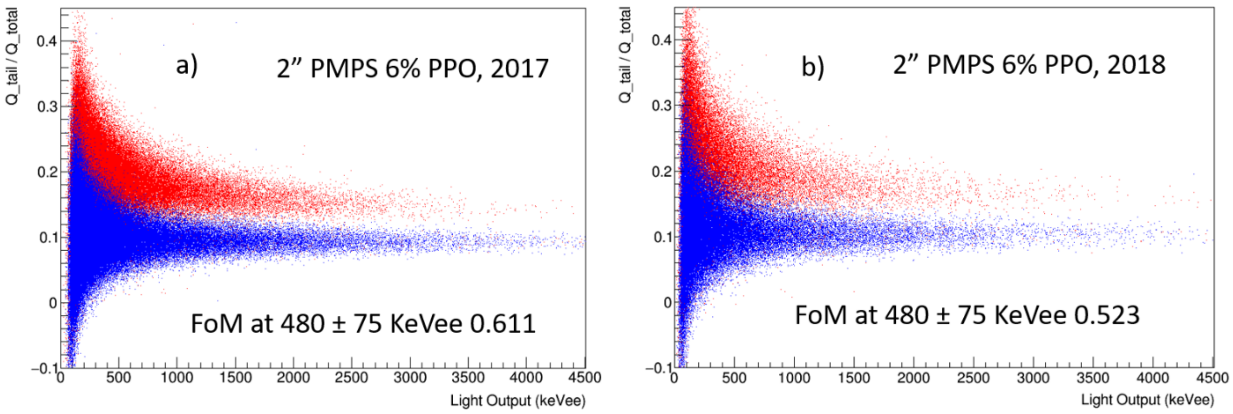

Figure S3. Two-dimensional plot of the PSP versus light output for two samples with the same composition but different age of production: 2017 in (a), 2018 in (b). The FoM at the indicated threshold (480 keVee) is almost the same and the slightly better performance shown by the oldest sample can be referable to the smaller size and higher light yield of this scintillator, as reported in Table II of the manuscript.
